# Effect of intraoperative blood transfusion during maternal cesarean section on serum electrolytes and inflammatory response plus cellular immune response: A retrospective study

**DOI:** 10.1097/MD.0000000000038200

**Published:** 2024-05-24

**Authors:** Fan Xia, Pengrong Li

**Affiliations:** a Blood Transfusion Department, Yichang Central People’s Hospital, Yichang, Hubei, China; b Gynecology and Obstetrics, Yichang Maternity& Child Healthcare Hospital, Yichang, Hubei, China.

**Keywords:** cesarean section, cellular immune response, electrolytes, inflammatory response, intraoperative blood transfusion

## Abstract

Analyzing the effect of intraoperative autotransfusion on serum electrolytes, inflammatory response and cellular immune response in puerperae undergoing cesarean section. This study is a retrospective study of 60 women who underwent cesarean section in our hospital from January 2022 to January 2023. The subjects were divided into 2 groups according to the blood transfusion mode of the patients. The differences in blood transfusion volume, blood transfusion volume, serum electrolyte, inflammatory response, cellular immune function, coagulation function and prognosis were compared between the 2 groups. The intraoperative blood transfusion volume, postoperative feeding time, the activity time since getting out of bed, the time of physical recovery and hospital stay in the observation group were lower compared to those of the control group, but the intraoperative crystal infusion volume and the colloid infusion volume in the observation group were higher compared to those of the control group (*P* < .05). Ca2+ concentrations of the observation group and the control group were lower compared with those of their same groups before surgery (*P < *.05), however, there were no statistically significant differences in the comparison of the Ca2+ concentrations between the observation group and the control group (*P* > .05). At 1d postoperatively, IL-1β, IL-6 and granulocyte-macrophage colony-stimulating factor (GM-CSF) were all higher (*P < *.05) and CD3+, CD4+ and CD4+/CD8+ were all lower (*P < *.05) in the observation group and the control group compared with those of their same groups before surgery. The IL-1 β, IL-6, and GM-CSF of the observation group were decreased compared to those of the control group (*P* < .05) and CD3+, CD4+, CD4+/CD8+ of the observation group were elevated compared to those of the control group (*P < *.05). Both autotransfusion and allogeneic blood transfusions during maternal cesarean section can attenuate the inflammatory response and have no significant inhibition of coagulation, and autotransfusion have less effect on the cellular immune response, are more effective in attenuating the inflammatory response, and significantly improve prognosis, although changes in Ca2+ concentration after transfusion require attention.

## 1. Introduction

Postpartum hemorrhage is the leading cause of maternal death and the most common reason for admission of the intensive care unit after surgery.^[1]^ A study showed that the prevalence of postpartum hemorrhage in China ranges from 0.62% to 0.93%.^[2]^ In addition, a foreign health report of a confidential survey of puerperae consistently found that hemorrhage was the most important direct cause of maternal death.^[3]^ Hemorrhagic puerperae undergoing cesarean section are at great risk, with the possibility of hemorrhagic shock and disseminated intravascular coagulation in the perioperative period, which not only increases the rate of hysterectomy but even threatens the life of the mother and the fetus. hemostasis and transfusion as soon as possible are important treatments.^[4]^

In these types of cesarean sections, allogeneic blood transfusions are often used to rapidly replace blood loss in response to postpartum hemorrhage. Allogeneic blood transfusion is also a common form of blood transfusion used in clinical practice today. Although allogeneic blood transfusions can relieve maternal blood loss, their clinical use is limited by the strict regulations on blood transfusion and the potential for adverse reactions such as allergy, hemolysis, fever, viral infection and immunosuppression.^[5,6]^

Intraoperative autotransfusion is an operation in which bleeding from the operating field is recovered, anticoagulated, filtered, washed, concentrated and then returned to the body. It belongs to the category of autotransfusion. The use of intraoperative or postoperative autotransfusion has expanded. Its use in surgery with small amounts of bleeding in healthy adults can modestly improve early postoperative Hb levels and tissue oxygenation, but has no significant effect on postoperative recovery. Patients who received autotransfusion had less suppression of cellular immune function and more rapid recovery of cellular immune function compared to allogeneic transfusions.^[7]^ It not only relieves the tight supply of blood (especially rare blood types), avoids the transmission of transfusion-related diseases and the hemolytic accidents caused by blood group incompatibility, but also buys time for resuscitation and reduces the medical costs incurred by transfusion. 2016 guidelines issued by the American Society of Anesthesiologists (ASA) and the Society for Obstetric Anesthesia and Perinatology clearly state that if there is insufficient blood in blood bank, or if the patient refuses to use banked blood, then use autologous blood for transfusing back.^[8]^ However, maternal blood is in a special physiological state of hypercoagulation in late pregnancy, and there are a number of theoretical and practical safety issues associated with autotransfusion in cesarean delivery. For example, Catling et al^[9]^ have shown that autotransfusion in obstetrics can lead to severe and life-threatening coagulation dysfunction. Based on this, the aim of this study was to analyze the effects of intraoperative autotransfusion on serum electrolytes, inflammatory response and cellular immune response in puerperae undergoing cesarean section.

## 2. Methodology

### 2.1. Study subjects

In this single-center retrospective study, we selected 60 women who underwent cesarean section in our hospital between January 2022 and January 2023. Patients were divided into a control group and an observation group according to different blood transfusion methods. The observation group received intraoperative autologous blood transfusion and the control group received intraoperative allogeneic blood transfusion. Inclusion criteria: American Society of Anesthesiologists classification I or II; Preoperative hemoglobin concentration (Hb) ≥ 110 g/L, hematocrit (Hct) > 34%, platelet count (Pit) ≥ 150 × 109; Normal coagulation function; Puerperae at high risk of bleeding, for example, placenta praevia, placental implantation, combined uterine fibroids in pregnancy, etc. Exclusion criteria: Preoperative combination of tumor, intrauterine infection, bacteremia, septicemia, sickle cell anemia; History of endocrine and immune disorders; Use of hormones and other drugs affecting immune function within 6 months before surgery; Severe liver and kidney dysfunction. Informed consent is signed by the puerpera and her family. The study was approved by the Ethics committee of Yichang First People Hospital.

### 2.2. Methodology

Preoperative routine monitoring usually includes ECG, blood pressure, respiration, SpO2 (PHILIPS MP30 monitor, The Netherlands), etc. Intra-vertebral block or general anesthesia was performed, with the intra-vertebral block anesthetic flat surface below the T4 level and the general anesthetic drugs such as propofol, remifentanil, sufentanil, and cisatracurium.

Intraoperative autologous blood recovery: Installing the piping system and blood recovery tank required for autologous blood transfusion as required, pre-fill the recovery tank and double-lumen suction tube with 100 to 200 mL of heparin water (heparin 12500U added to 500 mL of 0.9% sodium chloride solution), the ratio of dripping heparin water to recovered blood is 1:5,^[10]^ so that 1 mL of recovered blood contains 5U of heparin sodium, the number of heparin saline drops is adjusted at any time with the speed and volume of blood recovered. The blood is recovered from trauma bleeding and gauze aspiration into the blood storage tank by negative pressure suction and the negative pressure is controlled at 120 mm Hg to 150 mm Hg. When the blood collected in the recovery tank reaches 800 to 1000 mL or Hb ≤ 100 g/L, the automatic washing mode is activated. After filtration, washing, centrifugation, concentration and evacuation, impurities such as crushed red blood cells, anticoagulants, washing solutions and amniotic fluid components flow into the waste bag. The obtained concentrated red blood cells are pumped into the blood storage bag and transfused back into the body with the addition of a leucocyte filter according to the amount of intraoperative bleeding and vital signs, continuously implementing recovery-processing-recovery. Intraoperative bleeding is aspirated whenever possible and additional allogeneic red blood cell suspension and other blood components are added depending on the amount of intraoperative bleeding and the puerpera vital signs. In addition, a “bedside standby” strategy is used to improve cost-effectiveness by only using basic devices (blood recovery jars, suction tubes and anticoagulant solution) and using expensive materials (centrifuge cups, leucocyte filters) for blood processing only after sufficient recovered blood has been collected. The whole process is monitored by a trained and experienced anesthetist.

Timing of autotransfusion: If the bleeding volume exceeds 20% of the total body blood volume; For those who bleed <20% of the total body blood volume, the autologous blood should be transfused intravenously after the bleeding has basically stopped and the abdomen is closed.

Timing of allogeneic blood transfusion: 100 to 400 mL of fresh frozen plasma should be transfused intravenously when the Hb is 1.5 times normal; Platelets lO～40U should be transfused intravenously when the Plt is < 50 × 10^9^.

At the end of the operation, the inhalation and intravenous anesthetic drugs were stopped, the puerpera was taken to the recovery room to wait for her to resume spontaneous breathing, the tracheal tube was removed when the puerpera was sober, the puerpera continued to be observed until she reached the standard for discharge from the recovery room and was returned to the ward.

### 2.3. Evaluation indicators

Blood and fluid transfusion: Intraoperative blood loss, intraoperative blood transfusion, intraoperative crystalloid infusion and intraoperative colloid infusion were collected from these 2 groups. 5 mL of elbow venous blood was collected before surgery and 1d after surgery, and serum sodium (Na+), potassium (K+) and calcium (Ca2+) concentrations were measured by biochemical analyzer, and inflammatory factors [interleukin-1β (IL-1β), IL-6, serum granulocyte-macrophage colony-stimulating factor (GM-CSF)] and coagulation function indexes [thrombomodulin (TM), tissue-type fibrinogen activator (t-PA)]. The levels of cellular immune factors (CD3+, CD4+, CD8+) were measured by flow cytometry, and the levels of coagulation function indicators [D-dimer (D-D)] were measured by latex immunoturbidimetry. The prognosis of these 2 groups was compared by postoperative feeding time, the activity time since getting out of bed, the time of physical recovery, and the length of hospital stay.

### 2.4. Statistical analysis

SPSS 22.0 statistical software was applied to analyze the data. After the normality test, the measurement data were confirmed to the normal distribution and expressed as mean ± standard deviation by *t*-test, and the count data were expressed as percentages by Pearson chi-square test, Fisher exact test or Mann-Whitney test, with *P* < .05 indicating a statistically significant difference.

## 3. Results

### 3.1. General information

There was no statistically significant difference (*P* > .05) between the observation group and the control group when comparing the general information such as age, body mass index (BMI), number of pregnancies, number of deliveries and weeks of gestation. See Table 1.

**Table 1:** Comparison of general information between these 2 groups [n (%), x ± s].

Projects	The observation group (N = 30)	The control group (N = 30)	*t/χ*²	*P*
Age (yr)	29.75 ± 4.07	30.59 ± 2.78	0.933	.354
BMI (kg/m²)	26.32 ± 2.29	26.15 ± 1.93	0.306	.761
Pregnancy times (times)	4.26 ± 1.21	4.38 ± 1.46	0.340	.734
Number of outputs (times)	2.27 ± 0.29	2.17 ± 0.35	1.271	.208
Week of pregnancy (wk)	38.50 ± 0.68	38.57 ± 1.01	0.300	.765

### 3.2. Transfusion of blood and fluids

There was no statistical significance in the differences in the comparison between the intraoperative bleeding volume of allogeneic materials and those of the observation group (*P > .*05); The observation group had lower intraoperative blood transfusion, and higher intraoperative crystal transfusion and colloid transfusion than those of the control group (*P* < .05). See Table 2.

**Table 2:** Comparison in blood and fluid transfusions (x ± s).

Group	n	Intraoperative bleeding	Intraoperative blood transfusion	Intraoperative crystal infusion volume	Intraoperative colloid infusion volume
The observation group	30	1098.17 ± 288.78	317.82 ± 84.28*	1535.72 ± 311.24*	959.31 ± 329.61*
The control group	30	1122.24 ± 296.31	656.57 ± 168.43*	1294.31 ± 345.83*	649.63 ± 312.92*
*t*		0.319	9.852	2.842	3.732
*P*		.751	<.001	.006	<.001

* *P* < .05.

### 3.3. Comparison of serum electrolytes

Before surgery, the differences in Na+, K+ and Ca2+ concentrations between the observation group and the control group were not statistically significant (*P > *.05); 1d after surgery, the differences in Na+ and K+ concentrations between the observation group and the control group were not statistically significant (*P* > .05) compared with those of their same groups before surgery; Ca2+ concentrations in the observation group and the control group were lower (*P* < .05) compared with those of their same groups before surgery. However, there was no statistically significant difference in the Ca2+ concentration between the observation group and the control group (*P > *.05). See Table 3.

**Table 3:** Changes in serum electrolytes of these 2 groups (x ± s).

Projects	The observation group (N = 30)	The control group (N = 30)	*t*	*P*
Na+ (mmol/L)				
Preoperative	138.15 ± 3.43	137.82 ± 6.52	0.251	.802
1d after surgery	138.24 ± 4.22	138.65 ± 3.27	0.422	.674
K+ (mmol/L)				
Preoperative	3.79 ± 0.49	3.82 ± 0.34	0.297	.767
1d after surgery	3.81 ± 0.31	3.86 ± 0.48	0.451	.654
Ca2+ (mmol/L)				
Preoperative	2.12 ± 0.10	2.09 ± 0.11	1.116	.269
1d after surgery	1.63 ± 0.26*	1.61 ± 0.33*	0.269	.788

Compared with the preoperative situation,

* *P* < .05.

### 3.4. Comparison in inflammatory factors

**Figure 1.**
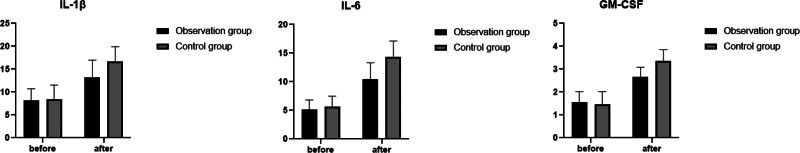
Changes in inflammatory factors.

Before surgery, there was no statistically significant difference in IL-1β, IL-6 and GM-CSF between the observation group and the control group (*P* > .05); 1d after surgery, IL-1β, IL-6 and GM-CSF were higher in both of the observation group and the control group compared with those of the same groups before surgery (*P* < .05), and IL-1β, IL-6 and GM-CSF were lower in the observation group compared with those of the control group (*P* < .05). See Table 4 and Figure 1.

**Table 4:** Comparison in inflammatory factors of these 2 groups (x ± s).

Projects	The observation group (N = 30)	The control group (N = 30)	*t*	*P*
IL-1β (ng/mL)				
Preoperative	8.24 ± 2.47	8.49 ± 3.05	0.343	.733
1d after surgery	13.15 ± 3.81*	16.73 ± 3.19*	3.948	<.001
IL-6 (pg/mL)				
Preoperative	5.16 ± 1.66	5.61 ± 1.86	0.989	.326
1d after surgery	10.41 ± 2.93*	14.38 ± 2.76*	5.400	<.001
GM-CSF (μg/L)				
Preoperative	1.56 ± 0.46	1.47 ± 0.55	0.675	.502
1d after surgery	2.67 ± 0.41*	3.37 ± 0.48*	6.096	<.001

Compared with the preoperative situation,

* *P* < .05.

### 3.5. Comparison in cellular immune factors

**Figure 2.**
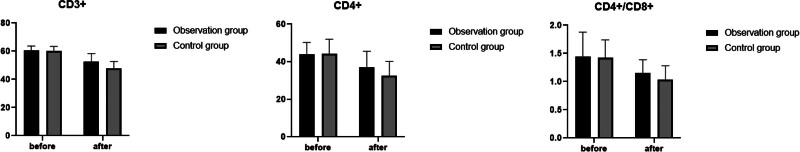
Changes in cellular immune factors.

Preoperatively, there was no statistically significant difference in CD3+, CD4+ and CD4+/CD8+ between the observation group and the control group (*P* > .05); 1d postoperatively, CD3+, CD4+ and CD4+/CD8+ were lower in both of the observation group and the control group compared with those of their same groups preoperatively (*P < *.05), and the CD3+, CD4+ and CD4+/CD8+ in the observation group were higher compared with those of the control group (*P < *.05). See Table 5 and Figure 2.

**Table 5:** Comparison in cellular immune factors of these 2 groups (x ± s).

Projects	The observation group (N = 30)	The control group (N = 30)	*t*	*P*
CD3+ (%)				
Preoperative	60.62 ± 3.08	60.16 ± 3.27	0.552	.582
1d after surgery	52.42 ± 5.91*	47.71 ± 5.01*	3.331	.001
CD4+ (%)				
Preoperative	43.82 ± 6.47	44.27 ± 7.69	0.243	.809
1d after surgery	37.04 ± 8.49*	32.52 ± 7.69*	2.162	.034
CD4+/CD8+				
Preoperative	1.45 ± 0.43	1.43 ± 0.31	0.213	.832
1d after surgery	1.16 ± 0.23*	1.03 ± 0.25*	2.138	.036

Compared with the preoperative situation,

* *P* < .05.

### 3.6. Comparison in coagulation function

Before surgery, the differences in the comparison of TM, D-D and t-PA levels between the observation group and the control group were not statistically significant (*P > .05);* 1d after surgery, the differences in TM, D-D and t-PA levels between the observation group and the control group compared with those of the same groups before surgery were not statistically significant (*P* > .05); The differences in TM, D-D and t-PA levels between the observation group and the control group compared with those of their same groups were not statistically significant (*P* > .05). See Table 6.

**Table 6:** Comparison in coagulation function of these 2 groups.

Projects	The observation group (N = 30)	The control group (N = 30)	*t*	*P*
TM (ng/L)				
Preoperative	27.41 ± 3.06	27.67 ± 3.96	0.288	.774
1d after surgery	28.76 ± 4.33	28.43 ± 4.60	0.277	.782
D-D (mg/L)				
Preoperative	3.55 ± 1.31	3.42 ± 1.23	0.384	.702
1d after surgery	3.67 ± 1.26	3.83 ± 1.22	0.507	.614
t-PA (μg/L)				
Preoperative	5.81 ± 1.39	5.77 ± 1.22	0.101	.919
1d after surgery	6.34 ± 1.12	6.19 ± 1.19	0.498	.620

Compared with the preoperative situation.

TM = thrombomodulin, t-PA = tissue-type fibrinogen activator.

### 3.7. Comparison in prognosis

The observation group had lower postoperative feeding time, activity time since getting out of bed, time of physical recovery and the length of hospital stay than those in the control group (*P* < .05). See Table 7.

**Table 7:** Comparison of prognosis in these 2 groups (x ± s).

Group	n	Postoperative feeding time (d)	Time out of bed (d)	Physical recovery time (w)	Length of stay in hospital (d)
The observation group	30	2.55 ± 0.61*	2.35 ± 0.81*	5.37 ± 1.06*	5.92 ± 0.57*
The control group	30	3.89 ± 1.27*	3.59 ± 1.42*	7.71 ± 3.75*	7.51 ± 3.10*
*t*		5.210	4.149	3.288	2.765
*P*		<.001	<.001	.001	.007

* *P* < .05.

## 4. Discussion

Hemorrhagic obstetric disease remains the leading cause of maternal death, accounting for 27.1% of maternal deaths.^[11,12]^ Common factors include placental implantation, placental abruption, placental vascular previa, weak contractions and abnormal coagulation. The surge in the number of surgical procedures has led to an increasing shortage of blood. How to use blood safely, reasonably, and effectively during the perioperative period has become an increasingly concerned topic.^[13]^ Allogeneic blood transfusion had been a routine clinical practice in the past, however, allogeneic blood transfusion is inevitably associated with acute lung injury, infections from infectious diseases and immunosuppression. Autotransfusion not only provides patients with fully compatible blood and avoids the risks of allogeneic blood transfusion, but also reduces the cost of transfusion and saves blood supply.^[14]^ In recent years, more and more studies have shown the safety of intraoperative autotransfusion in surgery.^[15–18]^ The use of intraoperative autotransfusion has been increasingly popularized in clinical practice.

The results of this study showed that 1d after surgery, the differences in Na+ and K+ concentrations between the observation group and the control group were not statistically significant (*P > *.05) compared to those of their same groups before surgery; The Ca2+ concentrations in the observation group and the control group were lower (*P* < .05) compared to those of their same groups before surgery, however, the differences in Ca2+ concentrations between the observation group and the control group were not statistically significant (*P* > .05). This suggests that neither autotransfusion nor allogeneic blood transfusion will cause significant effects on serum electrolyte levels such as Na+ and K+, while Ca2+ concentrations showed more significant changes. This may be due to the involvement of calcium ions as coagulation factor IV in many parts of the coagulation process. In addition, the combination of large amounts of sodium citrate preparations and calcium in the stock blood used in puerperae with allogeneic transfusions may also affect serum Ca2+ concentrations.^[19]^ This also suggests the need for prompt administration of calcium supplements to correct hypocalcemia in cases of severe postpartum hemorrhage, whether autologous or allogeneic blood is used.

IL-6 is an important B-cell stimulating factor that stimulates NF-KB abnormal activation, nuclear translocation and regulation of gene transcription by binding to the KB site on the corresponding target gene, inducing neutrophils to produce large amounts of platelet-activating factors, intensifying the inflammatory response while causing hyperfibrinolysis and increasing the risk of secondary bleeding and hemolytic disease.^[20]^ IL-1β is an inflammatory factor secreted by monocytes and macrophages that dilates arterial blood vessels and plays an important role in ischemia-reperfusion injury.^[21]^ Under normal physiological conditions, the expression of IL-1β in human blood is extremely low, and when the blood-placental barrier is disrupted, inflammatory factors are activated, which can further increase the risk of disseminated intravascular coagulation through NF-KB cellular regulation of adhesion molecule gene expression.^[22]^ GM-CSF is a peptide hormone hematopoietic factor released from endothelial cells during the inflammatory response, which acts on immune cells such as lymphocytes and neutrophils to participate in the body immune response and promotes the growth, differentiation and proliferation of mononuclear macrophages, which can be used as an indicator of the inflammatory response.^[23]^

The results in this study showed that before surgery, the differences in IL-1β, IL-6 and GM-CSF between the observation group and the control group were not statistically significant (*P* > .05); 1d after surgery, IL-1β, IL-6 and GM-CSF were higher in both of the observation group and the control group compared with those of their same groups before surgery (*P < *.05), and the IL-1β, IL-6 and GM-CSF were lower in the observation group compared with those of the control group (*P* < .05). This suggests that postoperative inflammatory reactions existed in varying degrees in both groups and that intraoperative blood transfusion helped to reduce the inflammatory reactions, and that the improvement was more obvious with autotransfusion than with allogeneic blood transfusion. The reasons for this may be related to the presence of immunosuppression and transfusion rejection of allogeneic blood transfusion. In addition, by comparing the postoperative prognosis of the puerpera in the autotransfusion group with the puerpera of the allogeneic transfusion group, it was found that the postoperative feeding time, the activity time since getting out of bed and the time of physical recovery were significantly shorter in the autotransfusion group than those of the allogeneic transfusion group. Studies have shown that^[24]^ abnormally high levels of IL-6 and TNFα in the blood of post-trauma patients usually indicate a high risk of death and poor prognosis in clinical practice; Conversely, the rapid and effective clinical reduction of IL-6 and TNF-α levels can significantly improve the prognosis of patients’ recovery and improve survival. Vishwakarma et al^[25]^ found that the autotransfusion resulted in more stable perioperative vital signs are more stable. They concluded that the reduction of fluctuations in vital signs depended on the reduction of pro-inflammatory factors and the stability of hemodynamics. Guo F et al^[26]^ also found that CRP, TNF-α and IL-6 in the blood of puerperae in the autotransfusion group were higher than those before the transfusion, but still significantly lower than those in the control group, suggesting that allogeneic blood transfusion inevitably involves even minor immune rejection, allowing inflammatory cells to infiltrate the vessel wall, and that the release of inflammatory factors affects the patient hemodynamic stability, thus exacerbating the inflammatory response. We know that surgery can lead to a stress response in the body that activates the release of inflammatory factors, and the immune capacity of the puerpera is suppressed after surgery. Li ZZ et al^[27]^ measured inflammatory factors in the blood of rats after autologous blood transfusion and showed that pro-inflammatory factors such as TNF-α, IL-6, IL-1β and IL-18 were suppressed in the blood of transfused autologous blood. The above study is consistent with the results of the present study. It is suggested that autotransfusion is conducive to reducing the inflammatory response of puerperae, reducing the incidence of postoperative infections and more beneficial for the postoperative recovery of puerperae.

It has been found that under normal physiological conditions, the coagulation and fibrinolytic systems maintain a dynamic equilibrium, while after device trauma, blood flow in the body slows down and blood-brain perfusion is affected, resulting in impaired microcirculation in brain tissue, coupled with the inevitable blood loss during delivery, the compensatory increase in red blood cells leads to increased platelet adhesion, aggregation and release, which can cause hypercoagulation.^[28]^ In addition, the coagulation system and fibrinolytic system are activated to varying degrees during the perioperative period, and the activation of the fibrinolytic system is reflected in increased D-D production.^[29]^ Therefore, it is essential to evaluate maternal coagulation after cesarean section. At 1d postoperatively, the TM, D-D and t-PA levels in the observation group and the control group were not statistically significant (*P > .*05) when compared with those of their same groups preoperatively; The TM, D-D and t-PA levels in the observation group and the control group were not statistically significant (*P* > .05). This suggests that autologous blood recovery did not have a significant effect on the coagulation function of the body.

CD3+ and CD4+ lymphocyte subpopulations are important indicators of cellular immune function; CD3+ reflects the overall level of cellular immune function, and its reduced level indicates suppressed cellular immune function; CD4+ plays an important role in recognizing foreign antigens, mediating the production of inflammatory factors and fighting infection, etc. Reduced CD4+ leads to the production of lymphokines and assists B lymphocytes in the production of antibodies, and assists the decreased function of other lymphocytes. The CD4+/CD8+ ratio is dynamically balanced and is a factor in the stability of the body immune function. A decrease in this ratio indicates an imbalance in the ratio of T-lymphocyte subsets and a decrease in cellular immunity. The neuroendocrine and immune systems form a complex 2-way regulatory network that works together to maintain the stability of the body internal environment. After trauma, surgery and infection, the body can stimulate immune cells and affected cells to produce a variety of cytokines that cause an inflammatory response and produce immunomodulatory effects.^[30]^ IL-6 is an important inflammatory factor in the neuroendocrine-immune system, and elevated levels of IL-6 can help improve the body specific immune function during inflammatory responses.^[31]^ Therefore, this study used these indicators to reflect the cellular immune function of the patients. The results of this study showed that CD3+, CD4+ levels and CD4+/CD8+ ratios were lower in the 2 groups of puerperae on the first postoperative day compared to those before the surgery, suggesting a decrease in the puerperae’ cellular immune function; The reason for this may be related to the absence of immunoneutral effectors in autologous blood.^[26,32,33]^

There are limitations to this study. The small sample size of the subjects included in this study resulted in the benefits of autotransfusion possibly not being fully realized. In addition, the bleeding volume was a clinical estimate and this, coupled with the small sample size, may have led to some bias in the results. Further sample size expansion is needed to further analyze the results of the study.

In summary, both autotransfusion and allogeneic blood transfusions during maternal cesarean section attenuate the inflammatory response and have no significant inhibition of coagulation, and autotransfusions have less effect on the cellular immune response, are more effective in attenuating the inflammatory response, and significantly improve prognosis, although changes in Ca2+ concentration after transfusion require attention.

## Author contributions

**Conceptualization:** Fan Xia, Pengrong Li

**Data curation:** Fan Xia, Pengrong Li

**Formal analysis:** Fan Xia, Pengrong Li

**Funding acquisition:** Fan Xia

**Investigation:** Fan Xia, Pengrong Li

**Methodology:** Fan Xia, Pengrong Li

**Supervision:** Fan Xia

**Writing – original draft:** Fan Xia, Pengrong Li

**Writing – review & editing:** Fan Xia, Pengrong Li
